# Effect of *Spirulina platensis* ingestion on the abnormal biochemical and oxidative stress parameters in the pancreas and liver of alloxan-induced diabetic rats

**DOI:** 10.1080/13880209.2017.1300820

**Published:** 2017-03-08

**Authors:** Ourida Aissaoui, Malek Amiali, Nora Bouzid, Khaled Belkacemi, Arezki Bitam

**Affiliations:** a Food Technology and Human Nutrition Laboratory, École Nationale Supérieure Agronomique (ENSA), Algiers, Algeria;; b Department of Anapathology, CHU Parnet, Algiers, Algeria;; c Department of Soil Sciences and Agri-Food Engineering, Université Laval, Quebec City, Quebec, Canada

**Keywords:** Antioxidant effect, phenolic compounds, phycobiliproteins, weight gain, organ weight, glycaemia, HOMA-IR, liver function, beta-cells, metformin

## Abstract

**Context:** Previous studies have shown that *Spirulina platensis* Gomont (Phormidiaceae) (*SP*) extract has beneficial effects on many disease conditions. The putative protective effects of *SP* were investigated in diabetic rats.

**Objective:** The current study investigates the antioxidant effects of *SP* in diabetic Wistar rats.

**Materials and methods:** Alloxan monohydrate (150 mg/kg body weight) was intraperitoneally administrated to induce diabetes. An aqueous suspension of *SP* powder in distillate water (10% w/v) was administrated orally by gavage (1 mL/day) for 50 days. Histopathological, biochemical and antioxidant analyses were performed. Glycemia, liver function and HOMA-IR were assessed using Spinreact and ELISA kits.

**Results:***SP* exhibited high-antioxidant activity. The IC_50_ values of the *SP* aqueous extract were 70.40 and 45.69 mg/L compared to those of the standard antioxidant BHT, which were 27.97 and 19.77 mg/L, for the DPPH and ABTS tests, respectively. The diabetic animals showed a significant increase in glycaemia (from 4.05 to 4.28 g/L) and thiobarbituric acid reactive substances (50.17 mmol/g protein) levels. Treatment with *SP* significantly reduced glycaemia by 79% and liver function markers [glutamate pyruvate transaminase (GPT), glutamate oxaloacetate transaminase (GOT) and alkaline phosphatase (Alk-p)]) by 25, 36 and 20%, respectively, compared to that of the controls. There was a significant increase in superoxide dismutase (48%), total antioxidant status (43%), glutathione peroxidase (37%) and glutathione reductase (16%) in the diabetic rats treated with *SP*.

**Discussion and conclusion:** These results showed that *SP* has high antioxidant activity, free radical scavenging, antihyperglycemic and hepatoprotective effects in diabetes.

## Introduction

In the past decade, the number of diabetes mellitus cases has gradually increased. This disease is the third leading cause of death worldwide, and the prevalence of diabetes for all age groups was estimated to be 2.8% in 2000 and is predicted to be 4.4% by 2030 (Wild et al. 2004). Diabetes mellitus is a multifactorial disease characterized by hyperglycaemia and increased basal metabolic rate (Bos & Agyemang 2013). In these conditions, the body does not produce sufficient insulin, a hormone produced by the β pancreatic cells. Insulin enables cells to absorb glucose and convert it into energy (Pankaj & Varma 2013). High blood glucose levels damage the cell membranes and generate reactive oxygen species (ROS) (Ha & Kim 1999). Despite the intensive effort to control this disease by pharmaceutical methods, antidiabetic drugs are still largely restricted due to their adverse effects and their expense (Zhang & Moller 2000; Oliveira et al. 2005; Abdel-Daim & Halawa 2014). Metformin is an antihyperglycemic drug used in the regulation of diabetes mellitus. To avoid the harmful side effects of chemical drugs, researchers have investigated natural products such as extracts from *Haematoccus pluviaris* Gomont (Haematococcaceae) and *Spirulina platensis* Gomont (Phormidiaceae) (*SP*), that possess antidiabetic effects and contribute to the nutrient requirements, stimulate the endocrine system and intermediate nutrient metabolism (Khan et al. 2005; Thormar 2012).

*SP* is a blue-green algae belonging to the cyanobacteria family that is rich in bioactive compounds, such as proteins, lipids, carbohydrates, trace elements (zinc, magnesium, manganese, selenium), pigments (phycocyanin, β-carotene), riboflavin, tocopherol and α-linoleic acid (Göksan & Kılıc¸ 2009; Yang & Zhang 2009; Yusuf et al. 2016). *SP* and its extracts are widely used as nutrients for human and animal consumption, natural dyes in food and cosmetics and nutraceutical and food additives for pharmaceutical industries (Zheng et al. 2013). Multiple studies worldwide have reported that *Spirulina* species can regulate diabetic processes, such as hypercholesterolemic activity, and have antioxidative effects and radical scavenging properties which provide significant multiorgan protection and ameliorate the effect of many drugs and chemicals-induced toxic assaults in laboratory animals (Pankaj & Varma 2013; Abdel-Daim 2014; Ibrahim & Abdel-Daim 2c015; Abdel-Daim et al. 2016). The major objective of this study was to determine the effects of *SP* on metabolic abnormalities, oxidative stress and histological changes in diabetic rats treated with alloxan.

## Materials and methods

### Phytochemical screening of *SP*

#### Determination of total polyphenols and flavonoids

An aqueous extract of *SP* (AE*_SP_*) was prepared in 2014 as described by Chu et al. (2010). Total polyphenols of AE*_SP_* were measured using the Folin–Ciocalteu method (Singleton et al. 1999); gallic acid was used as a standard for the calibration curve. The flavonoids of AE*_SP_* were measured using the AlCl_3_ method (Lamaison & Carnet 1990). Quercetin was used as a standard for the calibration curve.

#### Determination of phycobiliproteins and total carotenoids

*SP* powder (1 g) was suspended in 100 mL of sodium-phosphate buffer (0.1 M, pH 7.0, 1 mM sodium azide). The suspension was disrupted by sonication at 50 Hz for 60 s followed by freezing at −20 °C and thawing at room temperature (25 °C) in the dark. Extraction of the phycobiliproteins was performed by centrifuging the pretreated *SP* cell suspension at 10,000 *g* for 30 min at 4 °C. The dark-blue phycobiliprotein supernatant was collected, and the pellet was discarded. The absorbance of the phycocyanines (C-PC), allophycocyanines (APC) and phycoerythrines (PE) of the phycobiliprotein solution was measured using a SPECTRONIC® 20 GENESYS™ UV-Vis spectrophotometer (Virginia, USA) at wavelengths of 620, 652 and 562 nm, respectively (Anamika et al. 2005). 

For determination of the concentrations of C-PC, APC and PE, the following Equations (1–3) were used (Bennett & Bogorad 1973):

(1) $$\text{C}-\text{P}\text{C}\left(\frac{mg}{mL}\right)=\frac{\left[A620\;-\;0.474\;\left(A652\right)\right]}{5.34}$$

(2) $$\text{A}\text{P}\text{C}\left(\frac{mg}{mL}\right)=\frac{\left[A652-\;0.208\left(A620\right)\right]}{5.09}$$

(3) $$\text{P}\text{E}\left(\frac{mg}{mL}\right)=\frac{\left[A562-\;2.41\;\left(C-PC\right)\;–\;0.849\;\left(APC\right)\right]}{9.62}$$

Total carotenoids were also determined spectrophotometrically at 470 nm using the same UV/Vis spectrophotometer. The concentrations of chlorophyl a (Ca) and chlorophyl b (Cb) were determined at 653 and 666 nm, respectively. Total carotenoids and Ca and Cb levels were calculated based on Equations (4–6) (Lichtenthaler & Wellburn 1985).

(4) $$Ca=15.65\;*\;A_{666}\;-\;7.340\;*\;A_{653}$$

(5) $$Cb=27.05\;*\;A_{653}\;-\;11.21*A_{666}$$

(6) $$Total\;carotenoids=1000\;*\;A470\;-2.860\;*\;Ca-\frac{129.2\;*\;Cb}{245}$$

#### Determination of the antiradical and antioxidant activity and the IC_50_

The 2,2-diphenyl-1-picrylhydrazyl (DPPH) antiradical test was carried out as described by Burits & Bucar (2000). Butylated hydroxyl toluene (BHT) at 100 μg/mL was used as a positive control. The antiradical activity of the tested samples was calculated using the following equation (Equation 7):

(7) $$DPPH\;\left(\%\right)=\frac{1-A.s\;}{A.st}\;100$$

A.s and A.st are the absorbance of the tested samples and the DPPH, respectively.

The antioxidant activity assay is based on the ability of different fractions to scavenge 2, 2′-azino-bis (ethylbenzothiazoline-6-sulfonic acid: ABTS^. +^) (ABTS), a radical cation, compared to that of the standard (BHT). The antioxidative activity of the tested samples was calculated using the following equation (Equation 8) (Re et al. 1999):

(8) $$ABTS\;\left(\%\right)=\left(Ac-\frac{At}{Ac}\right)*100$$

At and Ac are the absorbance of the tested samples and ABTS^+^, respectively.

The extract concentration resulting in 50% inhibition (IC_50_) of DPPH, ABTS and the standard BHT was calculated from the graph plotting the inhibition percentages against the extract concentration. DPPH, ABTS and BHT were purchased from Sigma Aldrich GmbH, Sternheim, Germany.

### Animals

Adult male rats (Wistar strain; age: 10–13 weeks; weight: 180–200 g) were obtained from the Pasteur Institute of Algiers (Algeria). The rats were housed in polypropylene cages and maintained on a standard pellet diet (National Office for Food Livestock, Algiers, Algeria) with access to water *ad libitum* under standard conditions of temperature (24 ± 2 °C) and relative humidity (60–70%) with a 12 h light/dark cycle. All of the experimental procedures were approved by the Algerian Institutional Animal Care Committee, which belongs to the National Administration of Algerian Higher Education and Scientific Research (Algiers).

### Diabetes induction

Freshly prepared alloxan monohydrate [150 mg/kg body weight (b.w.)] purchased from Sigma-Aldrich Co. (USA) was intraperitoneally administrated to overnight-fasted rats (Kameswara Rao et al. 1999). Fructose was added to the drinking water to prevent hypoglycemic crisis. The same volume of sodium chloride (0.9% solution) was injected into the control rats. Fasting blood sugar (FBS) of the animals was measured after 72 h. Animals with an FBS level ≥2 g/L were considered diabetic.

### Experimental design

All of the animals were randomized and divided into six groups, with eight rats in each group, as described in Table 1. We distinguished four major steps, which were as follows: day 0 to day 10 (week of adaptation), day 10 to day 20 (diabetes induction by alloxan), day 20 to day 60 (treatment with *SP* and metformin), and day 60 to day 70 (therapy arrest).

**Table 1.** Experimental design.

		Time in days			
Groups	*N* = 8 rats	0–10	10–20	20–60	60–70
1	Normal control (NC)	week of adaptation	Induction of diabetes for groups: 2,4,5	Ingestion of SP for Groups 3, 5 and Met for Groups 4,6	Stopped SP and Met treatments
2	Alloxan without treatment (DC)				
3	*S. platensis* (10%) (SPC)				
4	Alloxan + Metformin (500 mg/kg bw) (D + Met)				
5	Alloxan+*S. platensis* (10%) (D + SP)				
6	Metformin (500 mg/kg bw) (Met C)				

The *SP* powder was obtained from the Tractebel Consult Office in association with the University Center of Biotechnology Algae, Tchad in 2013. It was formulated as a standard quality spray-dried product that was part of a bulk production composed primarily of proteins (63.05%), carbohydrates (19%), lipids (4.48%), fibers (3%**)** and ash (3%). The *SP* and metformin (Met) powders were suspended in tap water and were administrated orally to each animal using a gavage needle once daily (1 mL).

### Blood collection and biochemical analysis

The animals fasted for 12 to 14 h. Then, blood samples were collected from the retro-orbital plexus every 10 days. The blood was centrifuged at 3500 rpm for 15 min at room temperature (25 °C), and the plasma was stored in dry tubes at −20 °C.

Glucose and liver function tests [glutamate pyruvate transaminase (GPT) and glutamate oxaloacetate transaminase (GOT) and alkaline phosphatase (Alk-p)] were performed using SPINREACT diagnostic kits (UAA Ctra, Santa Coloma 7 E 17176 SantEsteve de BAS (GI), Spain) by the automated Random Access Clinical Analyzer PICTUS 200-DIATRON based on the colorimetric method.

To determine whether *SP* could stimulate the release of insulin, we measured serum insulin levels after the arrest of treatment with *SP* for 10 days (at day 70) using an ELISA kit (Boehringer Manheim Diagnostic, Mannheim, Germany). Because abnormalities in insulin activity are poorly detected by a single determination of glucose or insulin levels (Laakso 1993; American Diabetes Association 1998), insulin resistance was evaluated by the homeostasis model assessment estimate of insulin resistance (HOMA-IR) (Matthews et al. 1985; Haffner et al. 2002) as follows (Equation 9):

(9) $$\begin{array}{l} HOMA\;-\;IR\\ =\frac{fasting\;insulin\;level\;\left(\frac{\mu U}{mL}\right)\times \;fasting\;blood\;glucose\;\left(\frac{mmol}{L}\right)}{22.5} \end{array}$$

The antioxidant enzyme activities were determined using commercial kits (Randox Laboratories Antrim, UK) for superoxide dismutase (SOD), glutathione peroxidase (GPx), and glutathione reductase (GRx) and total antioxidant status (TAS). The serum TBARS levels were measured as described by Quintanilha et al. (1982).

### Organo-somatic index (OSI) and body weight gain (BWG)

Each animal was weighted every 10 days and was sacrificed after 70 days of treatment, and the organs were removed and weighed.

(10) $$OSI=\frac{Organ\;weight\;}{Total\;BW}\;100$$

(11) BWG (g)=Final BW-Initial BW

### Histopathological analysis

The pancreas and liver were fixed in 10% formaldehyde. After they were embedded in paraffin, they were cut into 3 μm sections and stained with haematoxylin and eosin (H & E) (Gomeri 1950). The sections were examined under a light microscope (Leica, Leica Store Miami, Coral Gables, FL) equipped with a camera system (Canon, Tokyo, Japan).

### Statistics and data processing

The results are expressed as the mean ± standard deviation (SD). The statistical analyses of the data were conducted using Microsoft Excel software (Microsoft 1 Excel 2010). The statistical significance between the means was analyzed using a Student Test of ANOVA (*t*-test) from Excel version 2010 (Microsoft Corporation, USA). 

## Results

### Phytochemical screening

*Spirulina* showed 5.54 ± 0.41 mg EAG/g extract of total polyphenols and 1.82 ± 0.05 mg EQ/g extract of total flavonoids. Phenolic compounds act as scavengers of free radicals; they play a major role in antioxidant activity and in stabilizing lipid oxidation as reported by many studies (Gezer et al. 2006; Turkoglu et al. 2007).

In this study, we also evaluated the major antioxidant composition of phycobiliproteins and carotenoids from *SP*. The values obtained for C-PC, PE and APC were 16.54 ± 0.12, 1.34 ± 0.08 and 2.06 ± 0.11%, respectively. The total carotenoid was 3.80 ± 0.20 mg/L.

Free radical scavenging is the general mechanism for antioxidants that inhibit lipid peroxidation in a relatively short time. *SP* showed high antioxidant activity as determined by the DPPH and ABTS tests. The ABTS test showed higher antioxidant activity than the DPPH test (70.19 and 64.99%, respectively) compared to that of BHT (77.07 and 73.15%, respectively) (Table 2).

**Table 2.** Antioxidant activities of *S. platensis* extract water.

Antioxidant activity (%) in 100 μg/mL				IC50 (mg/L)			
DPPH	BHT	ABTS	BHT	DPPH	BHT	ABTS	BHT
64.99 ± 0.20	73.15 ± 0.85	70.19 ± 0.14	77.07 ± 0.74	70.40 ± 0.76	27.97 ± 0.92	45.69 ± 0.75	19.77 ± 0.27

DPPH: 2-2-Diphenyl-1-picrylhydrazyl, Butyl hydroxytoluene, ABTS: 2.2Azino-bis (3-ethylbenzthiazoline-6-sulphonic acid), IC50: extracts concentration providing 50% inhibition.

### Relative BWG and organ weight

There was a significant decrease (*p* < 0.001) in the BWG in groups 2 and 4 (−16.92; −16.83 g, respectively) compared to that of the controls (+55.78 g) as shown in Table 3. There was a smaller (*p* < 0.001) weight gain in G5 (+30.13 g) compared to that of the controls, while a similar BWG was observed in G3 (42.42 g) for the rats treated with *SP*. Notably, *SP* treatment outperformed the Met treatment in alloxan-treated rats (G5 vs. G4). Similarly, the relative organ weight was monitored. We noticed a significant increase (*p* < 0.001) in the OSI (liver, left kidney) in groups 2 and 4 compared to that of the untreated group. However, the diabetic group treated with *SP* did not show any substantial changes.

**Table 3.** Effects of *S. platensis* on body weight, body weight gain, and relative weight liver and left kidney [% body weight].

	Rats		Liver	Left kidney	
Groups	Body weight gain [g]	Weight [g]	Relative weight	Weight [g]	Relative weight
G 1: NC	55.78 ± 5.31	7.08 ± 0.22	2.92 ± 0.14	0.91 ± 0.09	0.37 ± 0.03
G 2: DC	−16.92 ± 7.43***	5.06 ± 0.27***	3.08 ± 0.17	1.26 ± 0.13**	0.77 ± 0.10***
G 3: SPC	+42.42 ± 3.93	6.78 ± 0.40	3.00 ± 0.16	0.95 ± 0.06	0.42 ± 0.03
G 4: D + M	−16.83 ± 4.96	5.33 ± 0.51	3.03 ± 0.22	1.32 ± 0.55	0.75 ± 0.31
G5: D + SP	+30.13 ± 4.35‡	5.8 ± 0.63‡	2.62 ± 0.25†	1.17 ± 0.34	0.53 ± 0.16†
G 6: MetC	+52.33 ± 6.53	7.18 ± 0.47	2.96 ± 0.26	0.82 ± 0.12	0.38 ± 0.04

NC: Normal Control; DC: Diabetic Control; SPC: *SP* control; D + Met: Diabetic rats treated with metformin; D + SP: Diabetic rats treated with SP; MetC: Metformin Control. Each value represents mean ± SE (*n* = 8).

** *p* < 0.01, compared with group 1 values.

*** *p* < 0.001, compared with group 1 values.

† *p* < 0.01, compared with group 2 values.

‡ *p* < 0.001, compared with group 2 values.

### Biochemical parameters

#### Blood glucose level (BGL)

**Figure 1.**
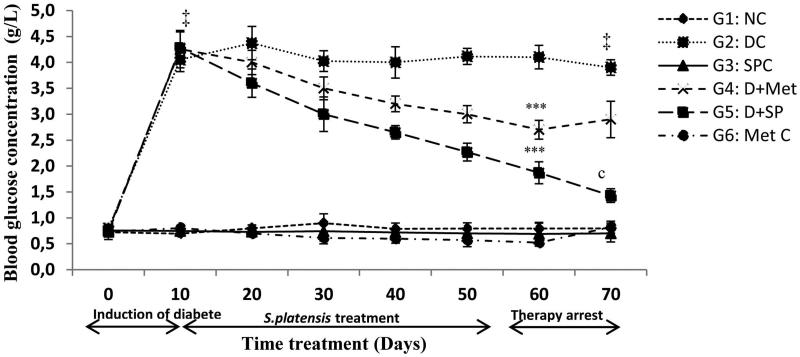
Effect of *S. platensis* administration on blood glucose level. NC: Normal Control; DC: Diabetic Control; SPC: *SP* control; D + Met: Diabetic rats treated with metformin; D + SP: Diabetic rats treated with SP; MetC: Metformin Control. Each value represents mean ± SE (*n* = 8). ‡*p* < 0.001, compared with group 1 values, ***p < 0.001, compared with group 2 values, (c) *p* < 0.001, compared the same group before and after therapy arrest (60th and 70th day) values.

**Figure 2.**
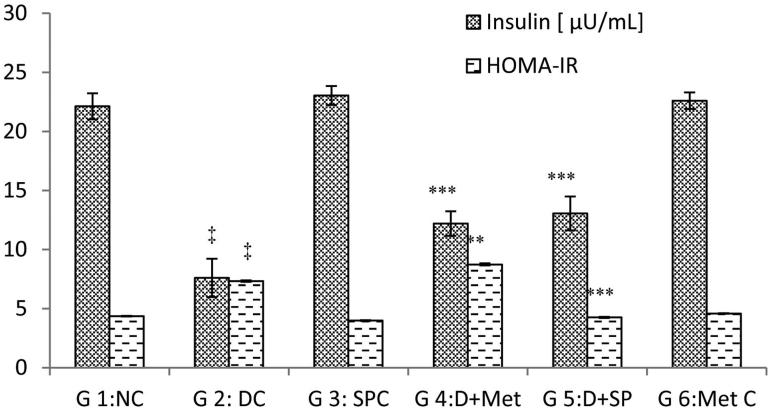
Effect of *S. platensis* administration on blood insulin level and HOMA-IR. NC: Normal Control; DC: Diabetic Control; SPC: *SP* control; D + Met: Diabetic rats treated with metformin; D + SP: Diabetic rats treated with SP; Met C: Metformin Control. Each value represents mean ± SE (*n* = 8). ‡*p* < 0.001, compared with group 1 values, *p < 0.05; **p < 0.01, ***p < 0.001 compared with group 2 values at the end of experiment.

The antihyperglycemic effect of *SP* in the diabetic rats induced by alloxan injections was indicated by an improvement in the fasting BGL, an important parameter for monitoring diabetes in addition to the plasma insulin levels as shown in Figures 1 and 2, respectively.

Hyperglycaemia induced by alloxan ranged from 4.05 to 4.28 g/L. *SP* treatment (G5) resulted in a significant 56% reduction (*p* < 0.001) in BGL, which varied from 4.28 to 1.87 g/L in the 50 days of therapy. The BGL of G4, varying from 4.26 to 2.70 g/L, decreased significantly (*p* < 0.001) by 36%. The BGL assessments were performed every 10 days over the 50 days of treatments. Ten days later, after stopping the treatment, the BGL was monitored again. In this case, the BGL of the diabetic-treated rats (G5) did not increase but continued to decrease by 23%. However, the diabetic rats in G4, which were treated with Met, showed a non-significant increase (7%) (Figure 1).

Serum insulin levels decreased significantly (*p* < 0.001) to 7.65 μU/mL in the diabetic group compared to those of the normal control (22.12 μU/mL). However, *SP* and Met administration increased the levels (*p* < 0.001) to near normal values compared to those of G2 [41% (G5) against 37% (G4)] during the 70 days. In the normal treated rats, there was a slight increase compared to the controls in the insulin levels after treatment with *SP*. Thus, *SP* has potent antihyperglycemic activity. Furthermore, this alga enhanced insulin levels in diabetic rats.

#### Oxidative stress enzymes and liver function profile

The effect of *SP* on serum oxidative stress and hepatic function is shown in Table 4. Diabetes induced by alloxan decreased the oxidative stress parameters very significantly (*p* < 0.001) in comparison to those of the controls after 70 days. Administration of *SP* and Met significantly increased TAS (43%; *p* < 0.01 and 17%; *p* < 0.05), GRx (16%; *p* < 0.01 and 3%; *p* < 0.01), SOD (48%; *p* < 0.001and 25%; *p* < 0.01) and GPx (37%; *p* < 0.001 and 22%; *p* < 0.01) in groups 4 and 5, respectively. In contrast, the serum TBARS levels increased significantly (*p* < 0.001) in G2 compared to those G1. Administration of *SP* decreased the TBARS levels by 37% in G5, but no significant changes in the serum TBARS levels (5%) were observed in G4. The G2 rats showed a very significant (*p* < 0.001) increase in AST, ALT, and Alk-p after injection of alloxan. However, *SP* decreased these effects (*p* < 0.001) by 25, 36 and 20%, respectively.

**Table 4.** Effect of *S. platensis* on serum oxidative stress and liver function levels.

Parameters	G1:NC	G2:DC	G3:SPC	G4:D + Met	G5:D + Met	G6:MetC
Oxidative stress						
TAS [mmol/L]	1.08 ± 0.05	0.46 ± 0.07‡	1.32 ± 0.043	0.58 ± 0.08*	0.81 ± 0.11**	1.17 ± 0.065
SOD [U/mL]	62.06 ± 5.20	30.12 ± 6.35‡	80.3 ± 4.12	40.41 ± 6.5**	57.85 ± 8.2***	69.40 ± 5.00
Gpx [U/mL]	8.56 ± 0.62	4.11 ± 1.70‡	9.43 ± 0.83	5.31 ± 1.20***	6.52 ± 1.44***	8.91 ± 0.43
GRx [U/g protein]	24.32 ± 2.11	17.84 ± 4.11‡	26.52 ± 2.05	18.44 ± 3.13	21.17 ± 3.63**	25.81 ± 2.21
TBARS [mmol/g protein]	23.92 ± 2.34	50.17 ± 6.54‡	21.91 ± 2.21	47.56 ± 4.70	31.63 ± 5.7***	22.37 ± 2.51
Liver function [UI/L]						
AST 60d	94.95 ± 4.56	141.04 ± 6.73	74.34 ± 7.98	123.56 ± 8.62	112.05 ± 5.53	84.90 ± 5.95
70d	95.10 ± 4.43	140.91 ± 8.24***	72.63 ± 6.48	141.56 ± 6.21c	105.36 ± 5.84‡	87.66 ± 7.84
ALT 60d	53.00 ± 9.14	80.43 ± 11.96	47.74 ± 8.64	70.20 ± 12.86	58.50 ± 9.20	56.24 ± 8.78
70d	53.60 ± 7.26	78.75 ± 12.90***	46.80 ± 7.74	82.11 ± 14.88b	50.33 ± 9.40‡b	57.08 ± 7.14
Alk-p 60d	66.69 ± 5.51	103.02 ± 11.74	47.12 ± 7.30	94.60 ± 14.64	85.56 ± 12.86	66.80 ± 9.42
70d	68.01 ± 5.10	99.36 ± 13.70***	46.26 ± 8.28	100.18 ± 12.46	79.64 ± 11.54‡b	67.40 ± 12.24

NC: Normal Control; DC: Diabetic Control; SPC: *SP* control; D + Met: Diabetic rats treated with metformin; D + SP: Diabetic rats treated with SP; MetC: Metformin control. Each value represents mean ± SE (*n* = 8).Oxidative stress (‡*p* < 0.001, compared with group 1 values,*p < 0.05; **p < 0.01, *** *p* < 0.001 compared with group 2 values at the end of experiment). Liver function (**p < 0.01, compared with group 1 values, ***p < 0.001, compared with group 1 values, †*p* < 0.01, compared with group 2 values, ‡*p* < 0.001, compared with group 2 values at the end of experiment, (a) *p* < 0.05, (b) *p* < 0.01, (c) *p* < 0.001, compared the same group before and after arrest of treatment (60th and 70th day) values).

### Histopathology

**Figure 3.**
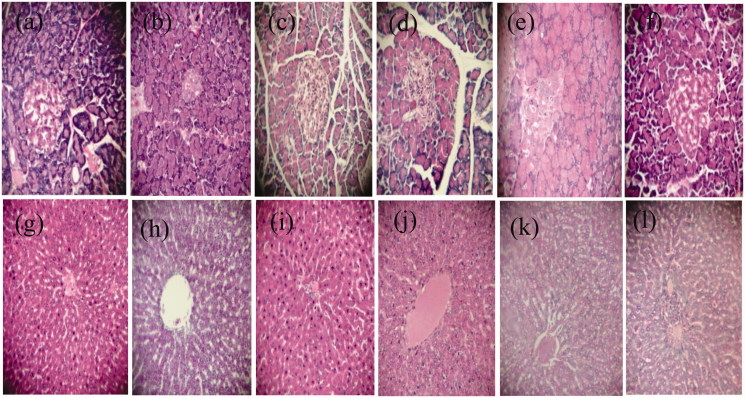
Effect of *Spirulina platensis* on the pancreas (a–f) and liver (g–l) of normal and diabetic rats H&E, 40×. Group 1 (a, g). Group 2 (b, h) Group 3 (c, i), Group 4 (d, j), Group 5 (e, k) and Group 6 (f, l).

Histopathological analyses of the pancreas (Figure 3(a–f)) and liver (Figure 3(g–l)) are shown in Figure 3. Normal rats (G1) had no structural changes in the pancreas. The pancreases of the diabetic rats (G2) revealed cell necrobiosis and a reduction in islet size. Marine *SP*-treated normal rats (G3) had large endocrine cells with granular cytoplasm, high eosinophil levels and clear vesicular nuclei, which indicate nucleoprotein synthesis (clumping of chromatin). Group 4 had small islets of Langerhans and a reduced number of cells with condensed nuclei with a partial restoration of the damage. However, after treatment with marine *SP* (G5), the diabetic rats showed a moderately size islets of Langerhans with active nuclei in the cells. This was likely due to an increase in the β-cells, leading to increased insulin production and secretion. Groups 1 and 3 showed normal liver parenchyma, while the liver sections of G2 showed primary degeneration of hepatocytes. Hypertrophy, hepatocyte necrosis and inflammatory infiltrates around the dilated centrilobular veins were observed. The liver sections obtained from the diabetic rats treated with metformin (G4) showed hypertrophy of hepatocytes and inflammatory infiltrates around the central veins and portal spaces, while the liver tissues of the diabetic rats treated with *SP* (G5) showed hepatocyte restoration with some hepatosteatosis.

## Discussion

Extensive studies have assessed the therapeutic benefits of *SP* on various disease conditions including diabetes, cardiovascular diseases and inflammatory diseases (Iyer et al. 2008; Juarez-Oropeza et al. 2009; Al-Dhabi 2013). *SP* is a unicellular cyanobacterium that has attracted attention for its high phytonutrient value; it has been consumed as a food for centuries and is currently used as nutraceutical food supplement worldwide (Deng & Chow 2010; Abdel-Daim et al. 2013).

Alloxan, a β-cytotoxin, induces ‘chemical diabetes’ in animal species by damaging the insulin-secreting cells of the pancreas and alters serum biochemical parameters. The major aim of this study was to assess the antidiabetic roles of *SP*.

The induction of diabetes by alloxan is associated with a progressive loss of body weight, which could be due to increased muscle wasting or excess breakdown of proteins in the tissues (Chatterjea & Shinde 2002). In contrast, oral administration of *SP* to diabetic rats for 50 days resulted in a significant (*p* < 0.001) increase in BWG, suggesting that *SP* substantially improved their general health status and metabolic mechanisms by effective glycaemic control or a reversal of gluconeogenesis (Voltarelli & de Mello 2008; Muthuraman et al. 2009; Abdel-Daim 2014; Yusuf et al. 2016).

The diabetic rats in Group 2 showed a considerable relative decrease in liver weight compared to that of the kidney. However, Yadav et al. (2005) and Sophia and Manoharan (2007) attributed the significant reduction in the liver weight of the diabetic animals to enhanced catabolic processes, such as glycogenolysis, lipolysis and proteolysis. The renal hypertrophy or increase in the kidney weight observed in the DC group may be due to increased glucose utilization and the subsequent enhancement in glycogen synthesis, lipogenesis and protein synthesis (Poonam et al. 2008; Vallon & Thomson 2012).

### Glycaemia and insulin

The injection of alloxan induces diabetes within 3 days (> 4 g/L). Oral administration of *SP* (10% w/v) restored the BGL to the normal range after 50 days. In this study, *SP* had a more potent effect than that of Met against diabetes as noted in groups 4 and 5. These results corroborate reports in the literature regarding diabetic rats treated with 10 mg/kg b.w. for 30 days and 25, 50 or 100 mg/kg p.o. of *SP* for 5 or 10 days after the alloxan injection (Muthuraman et al. 2009; Joventino et al. 2012). The antihyperglycemic effect of *SP* is believed to be due to either the presence of potent antioxidant bioactive molecules (β-carotene, phycocyanin and others), which increase the insulin secretion from the islet β-cells or promotion of blood glucose transport to the peripheral tissues (El-Baz et al. 2013). This antidiabetic effect could also be due to the action of peptides and polypeptides generated by the digestion of the *SP* proteins (Mani et al. 2000). Antioxidant effects of *SP* supplementation might be due to its high protein, essential amino acids, essential fatty acids, minerals, vitamins, carotenoids and other antioxidant active constituents, which promote growth and maintain health (Sanchez et al. 2003; Babadzhanov et al. 2004; Mata et al. 2010; Alvarenga et al. 2011; Abdel-Daim 2014).

In the last 10 days of the experiment, a significant reduction was observed (*p* < 0.001) in the BGL in Group 5. The strong antioxidant activity of the alga might also contribute to this effect by providing protection against the cytotoxic effects of the free radicals that are generated by the alloxan or diabetes itself (Gallo et al. 2005; Wadood et al. 2007).

Serum insulin levels decrease during diabetes. In the present study, we found that *SP* reversed the diabetic effects of glycemia and insulinemia. Similar results were reported by Muthuraman et al. (2009). The active fraction may exert antihyperglycemic effects in diabetic rats by increasing the pancreatic secretion of insulin from the existing β-cells. Bansal et al. (1981) reported that the increase in plasma insulin may be attributed to the conversion of proinsulin to insulin, possibly by pancreatic cathepsin B and/or its secretion.

### Oxidative stress

The increased TAS, SOD, GRx and GPx activities in the blood may be responsible for the inhibitory effect of *SP* upon alloxan-induced oxidative stress and the reduced lipid peroxidation level. These results were noted by Abdel-Daim et al. (2015). According to Shyam et al. (2007), supplementation with *SP* did not cause any significant change in the plasma total antioxidant status, although a trend toward higher values was evident, with the exception of the TBARS. Treatment with *SP* offered protection through attenuation of lipid peroxidation and decreased production of free-radical derivatives, as evident from the decreased levels of serum MDA and normalization of GSH and SOD levels (Abdel-Daim 2014).

This abnormal metabolism leads to an increased generation of ROS (Rajasekaran et al. 2006). The diabetogenic effect of alloxan is due to excess production of ROS. Excess ROS result in toxicity in the pancreatic cells, which reduces the synthesis and the release of insulin, and affects various organs, such as the liver, kidney, and the hematopoietic system (Sakurai et al. 2001; Sabu et al. 2002).

Diabetics and experimental animal models exhibit high oxidative stress due to persistent and chronic hyperglycaemia as well as hyperlipidaemia, which blocks the antioxidative defence system and thus promotes *de novo* free radical generation (Kamalakannan & Prince 2006; Vijayaraj et al. 2013).

Plant cell defences against the damaging effects of oxidative stress involve both enzymatic and non-enzymatic components. The enzymatic factors may directly scavenge ROS or, by producing a non-enzymatic antioxidant (as mentioned previously), may play an important role in the cellular response to oxidative stress by reducing certain ROS individually or in synergy (Candan & Tarhan 2003; Chen et al. 2008; Vo et al. 2015). *SP* supplementation enhanced all altered serum biochemical parameters and antioxidant biomarkers (Abdelkhalek et al. 2014).

### Liver function and histology

*SP* might play an important role in prevention and treatment of hepatic, renal and neurological disorders, especially those mediated by oxidative stress (Lu et al. 2010; Gad et al. 2011; Bhattacharyya & Mehta 2012; Abdel-Daim et al. 2016). In this study, alloxan injection was harmful and had a negative on the hepatic tissues, which was accompanied by an increase in GOT, GPT and Alk-p enzymes. GOT and GPT are cytosolic marker enzymes, reflecting hepatocellular necrosis (Setorki et al. 2010; Urmila et al. 2012). In contrast, the administration of *SP* had beneficial effects on hepatic balance and decreased liver function parameters significantly (*p* < 0.001) by 25, 36 and 20% for GOT, GPT and Alk-p, respectively, compared to those of DC 10 days after therapy arrest. *SP* dietary supplementation reduced the serum hepatic biomarkers and offered a good protection and maintained the structural integrity of hepatocellular membrane (Abdel-Daim 2014; Abdelkhalek et al. 2014). This may be due to the antioxidant activity of *Spirulina* phycobiliproteins (phycocyanins and allophycocyanins) or phenolic compounds (Nuhu 2013; Abdel-Daim et al. 2016), whereas the DPPH assay demonstrated that *SP* has free radical-scavenging activity. Therefore, this alga acts as an anti-hepatotoxicity agent (Abd El-Baky et al. 2009; Thomas & Kim 2011; Abdel-Daim 2014). A histopathological examination revealed that the *SP* supplement significantly improved the histological architecture of the islets of Langerhans and the liver of diabetic animals. Makhlouf and Makhlouf (2012), Tobon-Velasco et al. (2013) and Abdel-Daim et al. (2015) reported that the *SP* supplement had free radical scavenging activity and reduced various indicators of toxicity such as tissue damage in rats. *In vitro* and *in vivo* studies have shown that the antioxidant components produced by *SP* prevent or delay oxidative damage by reducing the accumulation of ROS (Zhang et al. 2011) through the activation of the antioxidant enzyme systems of catalase, SOD, and GPx (Thaakur & Jyothi 2007; Abdel-Daim 2014). In accordance with our results, it was proven that the antioxidant properties of *SP* contributed to its beneficial effect in treating various pathological conditions.

## Conclusion

*Spirulina* extract effectively alleviated the abnormal biochemical parameters, especially glycaemia and insulinemia. It may be used as a potent phytomedicine for diabetes alone or in combination with other treatments. *Spirulina* also reversed damage to the liver and the oxidative stress observed in diabetic animals. Furthermore, even if after therapy stopped, the *SP* efficacy maintained all of the studied parameters in a normal range, especially glycaemia. Other investigations are needed to elucidate the exact mechanism of action of the *SP* or its extract.
